# From diagnosis to intervention: a review of telemedicine’s role in skin cancer care

**DOI:** 10.1007/s00403-024-02884-7

**Published:** 2024-05-02

**Authors:** Kayla D. Mashoudy, Sofia M. Perez, Keyvan Nouri

**Affiliations:** 1 University of Miami Miller School of Medicine, 1600 NW 10th Ave #1140, Miami, FL 33136 USA; 2 Department of Dermatology and Cutaneous Surgery, University of Miami Miller School of Medicine, 1150 NW 14th Street, Miami, FL 33136 USA

**Keywords:** Skin cancer, Teledermoscopy, Cutaneous malignancy, Diagnostic accuracy, Mohs surgery, Artificial intelligence

## Abstract

Skin cancer treatment is a core aspect of dermatology that relies on accurate diagnosis and timely interventions. Teledermatology has emerged as a valuable asset across various stages of skin cancer care including triage, diagnosis, management, and surgical consultation. With the integration of traditional dermoscopy and store-and-forward technology, teledermatology facilitates the swift sharing of high-resolution images of suspicious skin lesions with consulting dermatologists all-over. Both live video conference and store-and-forward formats have played a pivotal role in bridging the care access gap between geographically isolated patients and dermatology providers. Notably, teledermatology demonstrates diagnostic accuracy rates that are often comparable to those achieved through traditional face-to-face consultations, underscoring its robust clinical utility. Technological advancements like artificial intelligence and reflectance confocal microscopy continue to enhance image quality and hold potential for increasing the diagnostic accuracy of virtual dermatologic care. While teledermatology serves as a valuable clinical tool for all patient populations including pediatric patients, it is not intended to fully replace in-person procedures like Mohs surgery and other necessary interventions. Nevertheless, its role in facilitating the evaluation of skin malignancies is gaining recognition within the dermatologic community and fostering high approval rates from patients due to its practicality and ability to provide timely access to specialized care.

## Introduction

Skin malignancy is one of the most frequently diagnosed cancers in the United States, where each man has a 1 in 37 lifetime risk and each woman has a 1 in 55 lifetime risk for developing a malignant melanoma [1]. In addition to the significant disease burden, estimates for treatment costs and value of life lost to society from melanoma alone vary from $8 billion per year to $15 billion per year [2]. The health and financial impacts of skin cancer are undeniably significant and increasing. It is ubiquitously known that early diagnosis and treatment of melanoma and nonmelanoma skin cancers improve patient outcomes and prognosis [3]. With the rising incidence of skin cancer cases, the demand for dermatologic care has increased as well as the wait time to see a specialist. Teledermatology has risen in popularity as a solution for these expanding challenges and offers an immediate and relatively thorough option for skin cancer evaluation.

The main types of virtual consultation are store-and-forward, allowing transmission of pictures and clinical details to a medical provider for review at a later time, and live interactive, which permits real-time interaction between the patient and physician. The combination of both styles yields a hybrid consultation [4]. Although the effectiveness of teledermatology for the diagnosis of skin cancer is multifactorial, studies have shown that it has acceptable accuracy and management concordance compared with in-person clinical evaluation, especially with the addition of dermoscopic images [5] and training of non-specialty providers giving remote dermatologic care [6]. Other important considerations to acknowledge are the enhanced practicality and convenience telemedicine provides for both patients and clinicians as well as the high satisfaction rate reported by many patients. Technologies like reflectance confocal microscopy and artificial intelligence are continuously being updated with the potential to improve the outcomes of remote-based care. Undoubtedly, establishing more teledermatology networks in medically underserved areas will improve access for geographically disadvantaged patents and may significantly lower the burden of cutaneous malignancies.

## Use of digital photography and dermoscopy in teledermatology

With the rapidly changing landscape of teledermatology over the past decade, new technologies have been introduced, and workflows have been updated to include these recent advances. Specifically, the integration of dermoscopy and mobile dermoscopy have supplemented the teledermatology experience, offering the potential to enhance virtual skin examination.

### Digital dermoscopy

In an online consultation, the touch and visualization aspects of an in-person visit are often replaced with digital photographs and dermoscopic images. These digital photographs not only strengthen the quality of the visit but also improve diagnostic accuracy for skin lesions [4]. Specifically, dermoscopy uses a lens-like device that allows for noninvasive magnified inspection of skin. Digital dermoscopy digitalizes and electronically transmits these images to providers. Initially, the images are captured with a digital dermatoscope or a digital camera affixed with a dermoscopic lens and saved within a secure web-based software [7]. Stentor (currently iSite), for example, is an online picture storing and communications platform used to archive and transmit digital dermatologic images. One publication examining the dermatology practice workflows of Kaiser Permanente Northern California found various advantages in using Stentor with dermoscopy. These benefits included greater probability of skin cancer detection compared with direct referral, lower probability of biopsy, fewer lesions requiring visits to the clinic, incorporation with electronic medical record (EMR), and long-term storage of images [8]. Thus, the addition of clinical and dermoscopic images has the potential to lower healthcare costs and boost skin cancer outcomes.

### Mobile dermoscopy

Many technologies are being employed to refine teledermatology practices and hold the capacity to improve skin self-examination. Particularly, mobile dermoscopy unites the photographic properties of mobile devices (smartphone, tablets, etc.) with their telecommunication features to take and send pictures of skin lesions. This innovation integrates attachable magnifying devices, mobile applications, and secure online platforms to support a detailed skin self-examination. These images, taken by either the patient, dermatologist, or primary care provider, can be transmitted straight to a clinician for remote evaluation and diagnosis [9]. Various algorithm-based softwares can even recognize characteristic features of some malignancies, but the applications with the highest sensitivity have images interpreted by a remote dermatologist [10]. Although they can be useful for melanoma screening, smartphone applications may not be diagnostically accurate unless the picture is examined by a skilled dermatologist and are not designed to conduct comparative analysis [11]. These points reaffirm that mobile dermoscopy serves as a supplement to regular full body skin examinations instead of a replacement. Supplying patient education on skin cancer, training patients’ partners in checking difficult-to-see areas, and fine-tuning algorithms to rule out benign skin lesions may improve the outcomes of this technology [9]. These and future advances in mobile dermoscopy continue to increase the reliability of skin self-examinations and confer earlier skin cancer detection.

### Imaging techniques and clinical utility of teledermoscopy

Various devices are available for dermoscopy including dermatoscopes with built-in digital cameras, dermatoscopes that attach to smartphones or mobile devices, digital single-lens reflex cameras, Apple mobile devices, digital full frame cameras, tablets, iPods, and smartphones. Some all-in-one platforms combine photo capturing, data archiving, and data transmitting properties [7]. The photographs taken with digital devices can be utilized and applied to clinical environments in a multitude of ways, including teledermoscopy, sequential monitoring, and machine learning [12]. 

The application of teledermoscopy to virtual dermatology consultations has also become widely used, particularly for large screening occasions and reaching rural or underserved areas that are distant from dermatology offices [13]. Moreover, inclusion of digital and dermoscopic photographs in teledermatology appointments has been shown to improve diagnostic accuracy with minimal offset to consultation time. Teledermoscopy’s strongest advantages, however, may reside in lowering healthcare costs, aiding patient triage and monitoring, and reducing the number of unnecessary referrals, surgery wait times, and “no-shows” for in-person appointments [14]. 

## Teledermatology for skin cancer consultation and documentation

### Triage

Skin cancer triage usually entails acting on clinical suspicion and employing dermoscopy to prioritize patients based on the urgency and stage of their skin lesions. Decisions may involve reassurance, biopsy, or referral to another specialist. Deciding on the most efficacious course of action is the priority during the triage process [15], and telemedicine’s role in facilitating these choices has also been investigate in various publications.

Since the consulting dermatologist has the patient’s clinical history and digital photographs at their disposal, the store-and-forward type of teledermatology enables timely management decisions [16]. In a study conducted at the University Hospital Virgen Macarena in Spain, the average wait time to see a dermatologist in person for a concerning skin lesion was 88.62 days as compared to 12.31 days via teledermatology [16]. Along with promptness, precision in management decisions is vital to fittingly organize patients according to their needs. Shapiro et al. examined 49 patients with both benign and malignant lesions and disclosed a 100% agreement rate among clinical dermatologists and teledermatologists on the decision to perform a biopsy [17]. Another investigation reported a 90% agreement rate among a clinical dermatologist and 3 teledermatologists regarding the management choices of 29 patients [18]. Comparing intra-dermatologist agreement rates among three teledermatologists evaluating 168 lesions, Whited et al. reported 69–70% concordance for medical therapy plans, 64–83% concordance for clinical therapy plans, and 59–69% concordance for diagnostic testing plans [19]. Thus, compared with clinic-based consultation, store-and-forward consultation provides relatively reliable management recommendations that may vary based on individual treatment preferences.

Although live video telehealth can also be employed for skin cancer triage, its multi-device and fast internet speed dependence render it a less popular option. The literature comparing live video conferencing to in-person consultation is also lacking. However, a recent study compared the management agreement (decision to biopsy or not) rates of compressed and uncompressed live video consultations to in-person consultations [20]. Of 214 patients evaluated, the mean proportion of cases recommended for biopsy was 0.04 for in-person consultation, 0.08 for uncompressed (higher resolution) virtual consultation, and 0.12 for compressed video virtual consultation. The higher propensity to biopsy for compressed (lower resolution) live video consultation indicates that the decision to biopsy is likely used as a safeguard, highlighting the importance of video quality in live video teledermatology.

### Diagnosis

As conveyed by the ABCDE criteria of melanoma diagnosis [21], current clinical approaches to skin cancer diagnosis largely depend on what the lesion looks like, supporting the integration of teledermatology due to its visual nature. The incorporation of dermoscopic images with teledermatology can further improve the visualization of sub-surface skin structures. In fact, dermoscopic features, such as black dots or hypopigmented areas, have specific histological correlates and distinguish dermoscopy as a highly sensitive and precise instrument for skin cancer detection [22]. Notably, teledermoscopy is a version of store and forward technology that transmits dermoscopic images for virtual consultation [23] and enhances the telehealth experience with high-quality images [24]. With appropriate training and access to a dermatoscope, primary care providers can also strengthen the reliability of their virtual dermatologic care. A recent paper in conjunction with the American Telemedicine Association (ATA) outlines specifications for the use of dermatoscopes in telemedicine [25]. These protocols and guidelines can help to standardize virtual dermatologic care across disciplines and the utilization of teledermoscopic images in diagnosing lesions.

In rural and underserved areas where caseloads outnumber specialists, teledermatopathology, a branch of telemedicine making pathologic diagnoses using digital histologic slides, is a budding mechanism for diagnostic care [26]. In addition to being time and cost effective by supplying a platform for prompt consultations, new evidence indicates that teledermatopathology might be just as effective as traditional face-to-face consultations in maintaining skin-related quality of life [27]. Despite these advantages, teledermatopathology has not been commonly adopted in the United States due to challenges like standardization of equipment, diagnostic auracy, licensure demands, and reimbursement strategies [26]. Regardless, much potential exists for teledermatopathology as both a primary source of diagnosis and as a vehicle for consultation or obtaining second opinions in cases where histopathologic expertise is warranted. These benefits are magnified in resource-limited areas as illustrated by collaborative projects that implemented teledermatology and teledermatopathology practices in Africa to bridge dermatological care gaps [28, 29]. 

## Treatment: surgical removal

### Teledermatology for preoperative consultation

According to a survey from the American College of Mohs Surgery, 67% of Mohs surgeons reported completing preoperative consultation with patients [30]. Consulting prior to surgery establishes an opportunity for the patient and surgeon to discuss the relative risks and benefits of the procedure. This appointment fosters patient comfort and better understanding of the surgical procedure [31]. Because this consultation is mainly informative, live video teledermatology can be implemented as a convenient and economic alternative. A retrospective analysis of Mohs surgery preoperative appointments within the Veterans Health Administration found that teledermatology consultations saved an average of 162.7 min, 144.5 miles, and $60.00 per person in average travel costs and significantly decreased consent failure rates for the surgery [32]. Teledermatology also decreased the wait time before surgery and increased the proportion of lesions treated within a 60-day period [32]. Telemedicine thus seems to be an effective modality for MMS preoperative consults and may improve the efficiency of subspecialty care.

### Teledermatology for post-surgical follow up

Due to the low risk of surgical site infection post Mohs surgery [33], the primary focus of a postoperative follow-up visit is to assess scar formation, wound healing, patient satisfaction, and answer any patient questions [33]. A randomized controlled trial of 90 patients undergoing Mohs surgery reported that patients preferred to receive wound care instructions via text messages for future visits and 91% of them found the service to be “helpful” or “very helpful.” [34] Thus, smartphone applications or postoperative text messaging may be beneficial in avoiding complications after dermatologic surgery and maximizing wound healing [35]. Postprocedural care and positive patient experience in the post-operative period is essential to dermatologic surgery outcomes, and telemedicine shows great potential for promoting optimal recovery in this period.

### Important considerations

#### Reliability and accuracy of teledermatology diagnosis and management

While teledermatology cannot completely replace the current gold standard of histopathological analysis, it produces acceptable rates of diagnostic accuracy, which continue to improve with imaging technology advancements. Most of the publications evaluating the effectiveness of teledermatology have reported accuracy rates of about 75–80% in comparison with in-person clinical care and 70% in comparison with histopathology [36]. According to the latest Cochrane systematic review on the use of teledermatology for skin cancer, sensitivity was about 95% and specificity was about 84% across studies using photographic images for diagnosing malignant skin lesions. For individual study estimates on using only dermoscopic images or a combination of dermoscopic and clinical images, reported sensitivities were similarly high but specificities were very variable for any skin cancer [37]. The sensitivities and specificities for the many reported diagnostic thresholds of invasive melanoma or atypical intraepidermal melanocytic variants were also inconsistent [37]. Another systematic review from 2011 concluded that the levels of diagnostic accuracy and concordance of both live video teledermatology and store-and-forward dermatology were acceptable in comparison to in-person care [38]. Comparing the diagnostic and management concordance between mobile dermoscopy and in-person evaluation, estimates from the few available studies are generally high, ranging from 81 to 91% [13], but smartphone applications with algorithm-based software are not as reliable in their diagnostic capabilities [39]. Overall, the success of teledermatology for diagnosing cancerous skin lesions is multifaceted, relying on variables like access to high-quality images, dermoscopic analysis, certain aspects of a patient’s clinical history, and the aptitude of the teledermatologist. Results from various studies investigating the diagnostic accuracy of teledermatology as compared to either in-person clincal diagnosis or histopathological diagnosis for cutaneous malignancies are included in Table 1 (Table 1).

Although the accuracy of a clinical diagnosis for cutaneous malignancies is vital, the suitability of clinical management has arguably more influence on patient outcome. For example, the decision to biopsy or excise a lesion may have greater implications for the criteria by which teledermatology should be evaluated. Interestingly, malpractice claims for skin cancer management via telemedicine are virtually nonexistent which may be partly due to high rates of appropriate management decisions [40]. In a study looking at the diagnostic and management accuracy of pigmented lesions in teledermatology, the rates of appropriate management plans for teledermatology were greater and/or equivalent to those of in-person evaluation for all lesions including benign ones [41]. However, for a subgroup of malignant lesions, management via teledermatology was significantly inferior to in-person dermatology such that up to one fifth of melanomas would have been improperly managed with teledermatology [41]. Therefore, clinicians should wary of the consequential limitations of telemedicine.

Recognizing that remote dermatological care is also routinely supplied by primary care providers, it is essential to investigate methods to reduce unnecessary referrals to the dermatology clinic. One Mayo Clinic investigation evaluated the impact of standardized templates on the referral patterns and dermatologic knowledge of family medicine providers. Using the standardized templates increased diagnostic and management concordance by 26.2% and 33.3%, respectively [6]. This pilot study illustrated that improvements in non-specialist virtual dermatological care can be accomplished through educational interventions. Thus, bestowing clinicians with a more extensive protocol for teledermatology workflow is advantageous and can be further strengthened with the application of dermoscopy practice guidelines [25]. 

### Patient satisfaction and barriers

For many patients who cannot drive or easily get to the dermatology clinic, teledermatology offers a relatively simple solution to gain access to care from their preferred setting. In particular, Whited et al. reported that most patients were confident in dermatologists using images during telemedicine visits to reach a diagnosis and viewed teledermatology consultations as more convenient than in-person appointments [42]. In two investigations in which patients submitted their own pictures either with or without dermoscopy, they noted being satisfied with the practicality and convenience [43] and reported willingness to pay out-of-pocket amounts for teledermatology services [44]. Other telehealth benefits appreciated by patients include more consistent skin monitoring, reduced waiting times, and enhanced privacy and comfort [5]. 

In addition to its many benefits, teledermatology holds possible barriers to its implementation in dermatology practices. As specified by a database study by Fogel et al., no reported cases with final findings of medical malpractice resulting from faulty management or negligence associated with teledermatology were found [40]. However, there may be non-reported malpractice cases, such as claims that are still being processed or were settled preceding a court decision, in addition to claims that have not yet been brought to light due to the common legal delay after a malpractice case has been established [40]. Dermatologists should be mindful of the potential malpractice risk that comes with the use of telemedicine and may want to limit the scope of their virtual visits to less concerning dermatologic conditions or expedite in-person visits for patients with atypical lesions [40]. Moreover, there is always a chance of acquiring low quality clinical, dermoscopic, or patient self-captured images that can make diagnosing via teledermatology unfeasible, as well as patients missing concerning lesions during skin self-examination [40]. The application of telemedicine to Mohs Surgery can also be challenging and poses its own limitations. Incorporating telemedicine into preoperative and postoperative surgical workflow could limit schedule flexibility for both the surgeon and the patient [45]. Another concern is the physical exam shortcomings imposed by teledermatology such that certain lesion characteristics like invasion depth may be difficult to accurately examine [45]. Patient skill and training with new technology may also be suboptimal given the initial learning curve and that the majority of skin cancer patients are elderly [45]. Certainly, the benefits of utilizing telemedicine must be measured against associated drawbacks and gauged with the unique framework of each practice in mind.

### Pediatric teledermatology

Beyond adult populations, teledermatology has arisen as a viable solution for pediatric patients obtaining dermatologic care in an environment rife with impediments to care. These barriers are multifactorial and are influenced by aspects related to a patient’s geographic location, caregivers and patients needing to miss work or school, caregivers needing to coordinate childcare for other children, lengthy wait times, insurance, and overall deficit of practicing pediatric dermatologists. Teledermatology programs present a promising solution to these problems and are most commonly utilized for evaluating conditions like atopic dermatitis, benign nevi, infantile hemangioma, inflammatory dermatoses, molluscum contagiosum, acne, and verruca vulgaris [46–48]. Studies have demonstrated a range of diagnostic concordance of pediatric telehealth with in-person evaluation between 70.1% and 89%, validating the success of these virtual visits for many dermatologic diseases [46]. 

Besides its diagnostic success, the utilization of telehealth for pediatric dermatology visits has been generally well-received among pediatric patients, parents, and dermatologists. Various papers have described high satisfaction rates ranging from 77 to 98.4% for this modality of dermatologic care [49–52]. Pediatric dermatologists, specifically have also expressed overall positive feedback regarding the application of telemedicine, with one study relaying that 90% of providers thought it would increase access to care, 77% predicted it could be time-efficient, and 69% believed it could be leveraged to sufficiently manage disease [51]. Nevertheless, physicians still have apprehensions about the quality of teledermatological care due to beliefs that more mistakes could be made, various technological issues during encounters, inability to perform in-office procedures, lack of examination by palpation, and challenges associated with assessing an actively moving child through video and keeping him or her engaged [46, 50]. Notably, the experiences of nurse practictioners and physician assistants with telehealth in pediatric patients has yet to be investigated and would be beneficial to explore in future studies.

### Future directions

With the rising popularity of teledermatology, many emerging technologies are being put into practice to enhance its experience. For instance, reflectance confocal microscopy (RCM), is a noninvasive examination technique that enables high-resolution in vivo assessment of skin lesions and shows promise for telemedicine settings [4]. Use of RCM in research and clinical environments has yielded high reliability and sensitivity in diagnosing both nonpigmented and pigmented lesions, but specificity greater depends on an individual’s’ training and skill level [53]. With the start of integration of RCM into clinical practice, educational programs for this technique are being further advanced to avoid misdiagnosis in both in-person and virtual settings. The application of RCM to telehealth learning platforms may complement the increasing knowledge base of clinical experts in addition to helping connect patients to trained providers [53]. 

Another technology with great potential for improving teledermatology efficiency is artificial intelligence (AI). Based on a meta-analysis of 70 studies, the accuracy of computer-aided diagnosis of melanoma was discovered to be comparable to that of clinical experts [54]. The dependability of AI may thus make it a complementary service in virtual consults by tracking lesions over time and expanding the differential diagnosis list [55]. Furthermore, the use of artificial intelligence as a telemedicine triage tool for patients with potentially malignant skin lesions can prioritize those who need expedited care [56]. In a study evaluating the application of AI in telemedicine triage and diagnosis of cutaneous lesions, Majidian et al. found no significant difference in diagnostic accuracy between a group of three dermatologists and AI using the software’s first three differential diagnoses [56]. 

However, the integration of two elaborate fields like dermatology and computational intelligence begets foundational challenges. For image-based technologies, data quality, quanitity, and diversity are conceivably the most influential factors of the model’s performance [57]. Efforts are underway to construct substantial open-source, representative, and continuously amended datasets that will be accessible by AI developers [58]. Another obstacle for AI development and implementation is the “black box” characteristic of modern machine learning such that the algorithm cannot explain its decicions-making rationale [59]. Explainable artifical intelligence (XAI) is an expanding field that has been proposed to overcome this limitation and commonly utilizes a “post hoc” approach for interpretation after the result is acquired, with more recent models adding attention visualization to the process [58]. Multimodal techniques incorporating inherently interpretable models, fine-grained structural heatmaps, and prototypical explanations are also being advanced for the realm of skin cancer recognition [60]. 

From a legal perspective, the lack of standardized explainability by these AI algorithms presents ongoing issues regarding potential unforeseen and perplexing failure forms. Medical AI may also be trained using subpar techniques, with incomplete data, or under improper conditions possibly leading to patient injury. Despite no case law on liability encompassing medical AI, physicians must take care to avoid medical malpractice liability by providing competent specialty-standard care and considering all accessible resources. Thus, under current legal codes, standard of care is intrumental to liability for medical AI [61]. Accompanying the Food and Drug Administration’s consideration of clinical decisions offered by machine intelligence as AI guided, a physician is held liable only when he or she does not comply with the legal standard of care and a patient injury follows [61, 62]. Accordingly, physicians should constantly gauge how to interpret AI recommendations and encourage administrative and legal efforts to develop guidelines for employing AI in specific clinical domains of need.

Overall, AI can still serve as a beneficial tool in recognizing lesions that require further workup and possible biopsy, especially for primary care providers or less experienced specialist providers [56]. Although AI is beneficial in triaging disease into broader categories, the expertise and contextual knowledge of dermatologists will always be needed for meticulous diagnosis, management recommendations, and atypical cases. Considering the rapid progression of technological developments, familiarization with the fundamental operations of AI along with its potential applications and current disadvantages will be crucial for the future of dermatology.

## Conclusion

Telemedicine is emerging as an indispensable facet of dermatology that offers enhanced access to specialty providers and money-saving solutions without compromising patient care quality. With the incorporation of technologies like mobile or digital dermoscopy, teledermatology can achieve excellent skin cancer detection rates with fewer biopsies, nonessential dermatology clinic referrals, and reduced wait times. Teledermatology can be provided through various styles including store-and-forward teledermatology, live video conferencing, and the combination of both types, called a hybrid consultation. With proper dermoscopy training and education, primary care providers can also upgrade the quality of their virtual dermatologic care. By strategically implementing telemedicine in the triage, diagnostic workup, and management of cutaneous malignancies, screening and treatment of these lesions may be expedited through refinement of clinical workflow. Moreover, teledermatology holds significant potential in promoting positive patient outcomes in the realm of preoperative and postprocedural care in Mohs micrographic surgery. Ongoing efforts center on increasing diagnostic accuracy through technological innovations in reflectance confocal microscopy and artificial intelligence. Overall, the many advantages of teledermatology including cost efficiency, reduction of travel burden, and acceptable accuracy rates in diagnosing skin cancer make it a widely employed clinical tool within the dermatology specialty.

**Table 1:** Diagnostic accuracy of teledermatology for skin cancer diagnosis

Source	Population	Design	Outcome
Warshaw et al., [38]	2152 patients: 2082 male, 70 female (mean age: 68 y)	Teledermatologist diagnosis using camera or dermoscopy taken images compared to in-person FTF* dermatologist diagnosis (Level: Benign vs. malignant)	Primary diagnosis agreement between TD** and FTF diagnosis: 45.7–75.7%
Borve et al., 2015, [63]	772 patients: 474 female, 298 male (mean age: 54 y)	Diagnosis as malignant or benign by 3 teledermatologists using dermoscopy images and clinical information on iDoc24 app compared with histopathological diagnosis (Level: Benign vs. malignant)	TD benign diagnosis agreed with Histopathology benign diagnosis: 99.1% TD malignant diagnosis agreed with Histopathology malignant diagnosis: 74%
Congalton et al., 2015, [64]	310 patients: 168 female, 142 male (mean age: 58 y)	Melanoma or non-melanoma diagnosis using MoleMapDiagnose software compared with histological diagnosis (Level: Malignant melanocytic vs. benign)	PPV*** of teledermatoscopic diagnosis of melanoma and positive histology was 63%
Borve et al., 2013, [65]	62 patients: 24 female, 38 male (mean age: 64 y)	Interobserver concordance between 2 teledermoscopists and a dermatologist using dermoscopy images and clinical information on iDoc24 smartphone app and FTF diagnosis compared with histopathological diagnosis (Level: Primary diagnosis)	Teledermoscopic diagnostic accuracy was 50.7% (teledermatologist 1) and 60.9% (teledermatologist 2) as compared with histopathological diagnosis FTF diagnostic accuracy was 66.7% as compared with histopathological diagnosis
Borve et al., 2013, [65]	40 patients: 23 female, 17 male (mean age: 49 y)	Diagnosis by dermatologists using images taken by GP**** from a mobile phone camera compared with FTF diagnosis by the same dermatologists (Level: Benign vs. malignant)	TD diagnosis agreed with FTF diagnosis: 31/40 patients; 78%
Boyce et al., 2011, [43]	55 patients: 22 female, 33 male (mean age: 26 y)	Dermatologist remote diagnosis using patient-generated images taken by mobile phones compared with traditional FTF diagnosis (Level: Primary management outcome – immediate action required, follow-up in 3 months, or no further action)	Exact diagnostic agreement in 116/167 analyzed lesions (69%) between remote diagnosis of patient-generated clinical images and FTF diagnosis
Warshaw et al., [41]	542 patients: 23 female, 519 male (mean age: 66 y)	Store-and-forward teledermatology diagnosis and FTF dermatologist diagnosis compared with histopathology diagnosis (Level: Primary diagnosis)	TD diagnostic agreement with histopathology diagnosis: 67% FTF diagnostic agreement with histopathology diagnosis: 81%

*FTF signifies face to Face

**TD signifies Teledermatology

***PPV signifies positive predictive value

**** GP signifies General Practitioner

## Data Availability

No datasets were generated or analysed during the current study.
